# Genistein Co-Amorphous Systems with Amino Acids: An Investigation into Enhanced Solubility and Biological Activity

**DOI:** 10.3390/pharmaceutics15122653

**Published:** 2023-11-21

**Authors:** Ewa Garbiec, Natalia Rosiak, Przemysław Zalewski, Lidia Tajber, Judyta Cielecka-Piontek

**Affiliations:** 1 Department of Pharmacognosy and Biomaterials, Poznan University of Medical Sciences, 3 Rokietnicka St., 60-806 Poznan, Poland; ewa.garbiec@student.ump.edu.pl (E.G.); nrosiak@ump.edu.pl (N.R.); pzalewski@ump.edu.pl (P.Z.); 2 School of Pharmacy and Pharmaceutical Sciences, Trinity College Dublin, University of Dublin, D02 PN40 Dublin, Ireland; ltajber@tcd.ie

**Keywords:** genistein, co-amorphous, amino acids

## Abstract

Genistein, an isoflavone known for its antioxidant and antidiabetic effects, suffers from the drawback of low solubility. To overcome this limitation, co-amorphous systems were synthesized by incorporating amino acids that were chosen through computational methods. The confirmation of the amorphous state of lysine and arginine-containing systems was ascertained by X-ray powder diffraction. Subsequently, the characterization of these systems was extended by employing thermo-gravimetry, differential scanning calorimetry, Fourier-transform infrared spectroscopy, and scanning electron microscopy. The investigation also included an assessment of the physical stability of the samples during storage. The apparent solubility of the systems was studied in an aqueous medium. To evaluate the in vitro permeability through the gastrointestinal tract, the parallel artificial membrane permeability assay was employed. The biological properties of the systems were assessed with regard to their antioxidant activity using 2,2-diphenyl-1-picrylhydrazyl and cupric ion-reducing antioxidant capacity assays, as well as their ability to inhibit α-glucosidase. The systems’ glass transition temperatures were determined, and their homogeneity confirmed via differential scanning calorimetry analysis, while Fourier-transform infrared spectroscopy analysis provided data on molecular interactions. Stability was maintained for the entire 6-month storage duration. The co-amorphous system containing lysine displayed the most pronounced apparent solubility improvement, as well as a significant enhancement in antioxidant activity. Notably, both systems demonstrated superior α-glucosidase inhibition relative to acarbose, a standard drug for managing type 2 diabetes. The results indicate that co-amorphous systems with lysine and arginine have the potential to significantly enhance the solubility and biological activity of genistein.

## 1. Introduction

Genistein (GEN) is an isoflavone found mainly in plants of the *Fabaceae* family [1], and in the human diet, its main sources are soy and soy products [2]. GEN is primarily known for its estrogenic properties [3,4], alleviation of menopausal symptoms [5,6], and reduction of the risk of some types of cancer, such as endometrial [7] and breast cancer [8]. However, there is growing evidence of GEN’s potential benefits in other diseases and disorders. Studies have shown its neuroprotective [9] and nephroprotective [10] effect. It may have a positive impact on some parameters associated with increased risk of cardiovascular disease [11] and modulate genes involved in the inflammatory process, thus being potentially beneficial in the control of psoriasis [12]. GEN also has anti-inflammatory and antioxidant properties [13] and has been reported as a promising compound to prevent and treat diabetes [14,15].

However, many of the above activity studies have been conducted in vitro at concentrations of GEN that may be difficult to achieve in human circulation due to its low bioavailability. Xu et al. reported GEN plasma concentrations only to be slightly above 5 µmol·L^−1^ at 24 h following oral administration of three doses of 10.3 µmol·kg^−1^ soy isoflavones [16]. According to the Biopharmaceutics Classification System, the main factor limiting the absorption of GEN is its low solubility. Therefore, this compound falls into the class II of this system due to its low solubility but high permeability [17].

To fully exploit the potential of GEN, attempts have been made to increase its solubility by the preparation of cocrystals [18] and complexes with κ-carrageenan [19]. Xiao et al. [20] investigated GEN encapsulated in zein nanoparticles with carboxymethyl chitosan coating. Fabricated nanoparticles provided sustained GEN release and improved antioxidant activity. Formulations also showed thermal and storage stability. These effects were particularly pronounced for biopolymer-coated and calcium ion-crosslinked nanoparticles. Some studies have focused on using other long-chain polymers, such as poly(ethylene glycol) (PEG) [17], Eudragit^®^ E100 [21], polyethylene glycol-polylactic acid (PEG-PLA) copolymers [22]. In the case of inclusion complexes of GEN with cyclodextrins studies have aimed at the use of alpha-cyclodextrin (α-CD), beta-cyclodextrin (β-CD), gamma-cyclodextrin (ɣ-CD), hydroxypropyl-β-cyclodextrin (HP-β-CD) and random methyl-beta-cyclodextrin (RAMEB-CD) [23,24]. With the exception of α-CD, the abovementioned cyclodextrins allowed GEN properties to be improved, in particular, ɣ-CD, HP-β-CD, and RAMEB-CD in terms of dissolution rate and ɣ-CD, HP-β-CD, and β-CD with respect to transport kinetics across the Caco-2 monolayer.

The solubility of a compound can also be improved by transformation to an amorphous state, which is characterized by long-range disorder and a higher energy state compared to its crystalline counterpart, making it possible to achieve high apparent solubility [25,26]. Some studies have confirmed that GEN can be rendered amorphous, and its solubility in such a form is improved. This has been achieved for GEN-loaded PEG microparticles [17], κ-carrageenan/GEN matrix [19] GEN micelles produced with PEG-PLA [22] and GEN inclusion complex using HP-β-CD and poloxamer 188 [27]. To date, an approach involving amino acids as co-formers to transform GEN into an amorphous dispersion by ball milling has not been reported in the literature.

Amino acids are organic compounds containing an amine and a carboxyl group, which allow them to form hydrogen bonds and interact with various molecules. They have been shown to be effective in stabilizing thermodynamically unstable (with a tendency to recrystallize) amorphous forms. The literature describes successful examples of co-amorphization of drugs with amino acids by various methods such as freeze-drying [28], spray drying [29,30], solvent evaporation [31], and grinding [32,33,34]. Stabilization of the amorphous compounds in such a co-amorphous system results from the physical separation of the molecules and/or formation of molecular interactions between the active pharmaceutical ingredient (API) and the co-former. A good co-former should allow stable amorphous systems with improved solubility to be obtained [35].

The aim of this work was: (i) to obtain and characterize co-amorphous systems of GEN and amino acids, (ii) to select the best co-former for GEN based on in-silico and experimental studies with respect to the ability to form a physically stable, co-amorphous system and (iii) to evaluate the improvement in physical properties with respect to solubility, permeability, and biological properties with regards to antioxidant activity and α-glucosidase inhibition.

## 2. Materials and Methods

### 2.1. Materials

GEN (purity ≥98%) was provided by Xi’an Tian Guangyuan Biotech Co. (Shenyang, China). Lysine (LYS; purity ≥98%), arginine (ARG; purity of 99%), α-D-glucopyranoside (PNPG), type I α-glucosidase from *Saccharomyces cerevisiae* in lyophilized powder form (≥10 units/mg protein), acarbose, 2,2-diphenyl-1-picrylhydrazyl, neocuproine (purity ≥98%) were supplied by Sigma-Aldrich (St. Louis, MO, USA). Tryptophan (TRP; purity >98.5%), tyrosine (TYR; purity >98%), glutamine (GLU; purity >98.5%) were delivered from TCI Chemicals (Portland, OR, USA), and ammonium acetate pure from Chempur (Piekary Śląskie, Poland). Methanol (HPLC grade), ethanol, and copper (II) chloride dihydrate pure were provided by POCH (Gliwice, Poland). Acetic acid, dimethyl sulfoxide, and sodium hydroxide were supplied by Avantor Performance Materials Poland S.A. (Gliwice, Poland). All reagents for the PAMPA assay (GIT lipid solution, Prisma HT, and Acceptor Sink Buffer) were purchased from pION (Forest Row, East Sussex, UK). Direct-Q 3 UV Merck Millipore (Burlington, MA, USA) purification system was utilized to prepare water of both high and ultra-high pure quality.

### 2.2. Molecular Modeling

The GEN and amino acid structures needed for molecular docking (MD) were obtained from the PubChem database in SDF format (website: https://pubchem.ncbi.nlm.nih.gov/, accessed on 10 November 2022). To prepare for MD, the structures were optimized using GaussView version E01 (Wallingford, CT, USA) software (B3LYP/6-31 (d,p)) and saved in MOL2 format. Open Babel (the Open Babel Package, version 2.4.0, http://openbabel.org, accessed on 15 November 2022) was then used to convert all MOL2 files to PDBQT format.

Subsequently, AutoDock Tools (ADT; Scripps Research Institute, La Jolla, San Diego, CA, USA) [36] was used to prepare the ligands and proteins. This involved calculating polar hydrogen atoms and Kollman and Gasteiger charges for the amino acid. Docking was then performed using Autodock Vina version 1.2.0 (The Scripps Research Institute, La Jolla, San Diego, CA, USA) [37,38], with default parameters for grid spacing and center grid box. The grid size was set to 40 × 40 × 40 grid points, resulting in 64.000 grid points on each map. The grid parameter file (GPF) format was used to save the output, which was then transformed into grid log files (GLG) using AutoGrid. The Lamarckian genetic algorithm was applied, and the output was saved in docking parameter file (DPF) format. After generating the DPF file, AutoDock was run, and the output was saved as a docking log file (DLG). This file contained data on the top ten free binding energies and RMSD values for GEN binding to the amino acid. The best scoring pose was selected and exported to the PDBQT format, which was then converted to PDB format using the Open Babel program. The PLIP server (https://plip-tool.biotec.tu-dresden.de/, accessed on 16 November 2022) [39] was used to determine the interactions between GEN and amino acids, and the results were visualized using the PyMOL tool (DeLano Scientific LLC, Palo Alto, CA, USA).

### 2.3. Preparation of Co-Amorphous Systems

#### Ball Milling

A total of 500 mg of physical mixtures containing the GEN and amino acid in a 1:1 molar ratio was milled in a ball mill Mixer Mill MM400 (Retsch GmbH, Haan, Germany) in 25 mL stainless steel milling jar containing two stainless steel balls with a diameter of 10 mm. The milling process was conducted at a frequency of 30 Hz for a cumulative duration of 90 min, which was divided into three milling cycles, each lasting 30 min, interspersed with 10 min breaks. Additionally, pure GEN was milled under the same conditions. Samples were kept in a desiccator that was protected from sunlight.

### 2.4. Identification of Genistein—Amino Acids Systems

#### 2.4.1. X-ray Powder Diffraction

All samples were investigated by X-ray powder diffraction using a Bruker AXS D2 Phaser diffractometer (Bruker, Germany), equipped with a CuKα X-ray radiation source (30 kV, 10 mA, λ = 1.54060 Å). Data were collected at ambient conditions, over a diffraction angle 2θ range of 5–45° with a step size of 0.02° and a counting rate of 2 s·step^−1^. The data were imaged and integrated with the Origin 2021b software (OriginLab Corporation, Northampton, MA, USA).

#### 2.4.2. Thermogravimetric and Differential Scanning Calorimetry

Thermogravimetric (TG) analysis was conducted using TG 209 F3 Tarsus^®^ micro-thermobalance (Netzsch, Selb, Germany). Approximately 6 mg of powdered samples were put into an alumina sample pan and subjected to a temperature increase of 10 °C per minute, starting from 25 °C and reaching 550 °C, while being exposed to a flow of nitrogen gas at a rate of 250 mL·min^−1^.

To perform a differential scanning calorimetry (DSC) analysis, a DSC 214 Polyma differential scanning calorimeter (Netzsch, Selb, Germany) was used. A blank aluminum DSC pan served as the reference sample, while sealed pans containing 9–10 mg of powdered samples with a hole in the lid were used. The melting point and glass transition temperature (Tg) of GEN were determined through a sequence of temperature changes. Initially, the sample was subjected to a temperature range from 25 °C to 325 °C with a consistent heating rate of 10 °C per minute. Following this, it was held isothermally at 325 °C for 10 min. In the subsequent step, the temperature decreased from 325 °C to 25 °C at a steady cooling rate of 40 °C per minute and then held isothermally at 25 °C for 2 min. Finally, the temperature was raised from 25 °C to 325 °C at a constant heating rate of 40 °C per minute.

Similarly, for the GEN-amino acid systems, the Tg was determined using a specific set of parameters. This involved an initial temperature range from 25 °C to 200 °C with a constant heating rate of 40 °C per minute. It was then kept isothermally at 200 °C for 5 min. In the next stage, the temperature was reduced from 200 °C to 25 °C at a constant cooling rate of 40 °C per minute and then held isothermally at 25 °C for 2 min. Finally, the temperature was raised from 25 °C to 200 °C at a constant heating rate of 40 °C per minute. The analysis was carried out under a nitrogen atmosphere with a flow rate of 250 mL·min^−1^.

The obtained TG and DSC data were analyzed using Proteus 8.0 (Netzsch in Selb, Germany) software, and the results were visualized using Origin 2021b software (OriginLab Corporation in Northampton, MA, USA).

The true density of GEN, ARG, and LYS required for the calculation has been established by utilizing a helium gas pycnometer (Accupyc 1340, Micrometrics Instrument Corporation, Norcross, GA, USA). To determine the density, the pressure variation of helium in the 10 cm^3^ sample chamber of the pycnometer was measured.

#### 2.4.3. Fourier-Transform Infrared Spectroscopy

An IRTracer-100 spectrophotometer with a diamond ATR module was used to measure spectra in the mid-infrared region, with a wave number range of 4000–400 cm^−1^, at a resolution of 4 cm^–1^ (400 scans). The LabSolution IR version 1.86 SP2 software (Warsaw, Poland) was utilized to acquire and process the spectra.

The Density Functional Theory (DFT) method, utilizing Becke’s B3LYP hybrid functional and 6-311G(d,p) basis set, was employed to optimize the molecular geometries of GEN and amino acids. Additionally, calculations were conducted for normal mode frequencies and intensities. The PL-Grid platform, accessed through the website www.plgrid.pl on 10 November 2022, was utilized for DFT calculations using the Gaussian 09 package (Wallingford, CT, USA). The GaussView version E01 (Wallingford, CT, USA) program was used to suggest the initial geometry of the molecules and for visual inspection of the normal modes. Data obtained from the calculations were analyzed using the Origin 2021b software (OriginLab Corporation, Northampton, MA, USA).

#### 2.4.4. Scanning Electron Microscopy

Microscopic observations of the initial and ball-milled powders were performed using a QUANTA 250 FEG (FEI, Eindhoven, The Netherlands) scanning electron microscope using a large-field detector and low accelerating voltage of 10 kV.

### 2.5. Physical Stability

The co-amorphous systems were stored under ambient conditions for six months. XRPD measurements were used to assess the recrystallization tendency of obtained systems every two weeks for the first two months and then once a month for the next four months after sample preparation.

### 2.6. Physicochemical Properties

Chromatographic Conditions

The analytical conditions for the GEN content analysis via HPLC were carried out following previously reported method [40], with modifications. The method was fully validated according to ICH guidelines. The validation parameters and chromatogram of GEN for the method that was developed are provided in Table S4 and Figure S12 (Supplementary Materials). The Shimadzu Nexera system (Shimadzu Corp., Kyoto, Japan) equipped with a diode array detector was applied. Samples were injected into Dr Maisch ReproSil-Pur Basic-C18 column (250 mm × 4.6 mm; 5 µm; Dr Maisch, Ammerbuch-Entringen, Germany). The injection volume was dependent upon the study, with 1 µL utilized for the apparent solubility assessment and 10 µL employed for both the powder dissolution and permeability evaluations. The temperature of the column was set to 30 °C. The mobile phase comprised a degassed mixture of 0.5% acetic acid and acetonitrile (35:65, *v*/*v*). The flow was isocratic at a flux of 1 mL·min^−1^, and the duration of the method was 7 min. The analysis wavelength was 260 nm.

#### 2.6.1. Apparent Solubility Studies

The solubility studies were carried out in distilled water. A total of 6 mg of crystalline GEN and a quantity of co-amorphous systems samples in excess of its expected saturated solubility were administered with 5 mL of a medium and placed in laboratory incubator MaxQ 4450 (Thermo Scientific, Waltham, MA, USA) maintained at 25 °C, agitation speed of 75 rpm and protected from light. After 2 h, the obtained solutions were filtered through a 0.45-μm nylon membrane syringe filter and injected into the HPLC system for GEN content analysis.

#### 2.6.2. Powder Dissolution Studies

Powder dissolution studies were conducted utilizing a paddle-equipped apparatus. The samples, consisting of either 10 mg of pure GEN or GEN-AAs co-amorphous systems equivalent to 10 mg of GEN, were sprinkled onto the surface of 500 mL of distilled water. The paddles were rotated at a speed of 75 rpm, and the temperature was maintained at 37 °C. At specified time points (5, 15, 30, 45, 60, 90, 120, and 180 min), a 2 mL aliquot of the sample was collected with the substitution of pre-heated dissolution medium and subsequently strained via a 0.45-μm nylon membrane syringe filter. The dissolved quantity of GEN was measured using a validated HPLC method.

#### 2.6.3. In Vitro Parallel Artificial Membrane Permeability Assay (PAMPA)

Gastrointestinal permeability through passive diffusion was evaluated in a cell-free permeation model. Samples of the GEN-AAs co-amorphous systems were prepared in the same way as for the solubility test, and GEN was dissolved in dimethyl sulfoxide. The experiment was performed in two 96-well plates. Each well of the donor plate was filled with a sample mixed with donor solution with pH adjusted to 6.8, and an Acceptor Sink Buffer was added to each well of the acceptor plate. The microfilter discs that separate the wells were impregnated with GIT-0 lipid. The acceptor plate was placed into the donor plate, creating a PAMPA sandwich, which was incubated for 3 h at 37 °C. The developed and validated HPLC method was used to analyze GEN concentrations, and the following equations were used to calculate the apparent permeability coefficient (*P_app_*):

$$Papp=\frac{-ln(1-\frac{C_{A}}{C_{equilibrium}})}{S\times \left(\frac{1}{V_{D}}+\frac{1}{V_{A}}\right)\times t}$$

$$C_{equilibrium}=\frac{C_{D}\times V_{D}+C_{A}\times V_{A}}{V_{D}+V_{A}}$$

where *C_A_* is the concentration in the acceptor well, *C_D_* is the concentration in the donor well, *V_D_* is the donor volume, *V_A_* is the acceptor volume, *S* is the membrane area, and *t* is the time (in seconds).

Substances found as low permeable have a *P_app_* below 0.1 × 10^−6^ cm·s^−1^, compounds considered to be medium permeable have 0.1 × 10^−6^ cm·s^−1^ ≤ *P_app_* < 1 × 10^−6^ cm·s^−1^, and those whose *P_app_* is ≥ 1 × 10^−6^ cm·s^−1^ are classified as highly permeable [41].

### 2.7. Biological Assays

#### 2.7.1. Antioxidant Activity Determination

##### DPPH Method

Antioxidant activity against 2,2-diphenyl-1-picrylhydrazyl radical was assessed according to the previously described method [42] with minor modifications, as follows: 25 μL of the sample was mixed with 175 μL of 0.2 mM DPPH solution, and incubated for 30 min at room temperature in the dark with shaking. Absorbance was measured at 517 nm using a UV/Vis microplate spectrophotometer (Multiskan GO, Thermo Fisher Scientific, Waltham, MA, USA). The control was composed of 25 μL of distilled water or dimethyl sulfoxide and 175 μL of DPPH solution.

##### CUPRAC Method

The antioxidant capacity of samples was investigated according to the previously described method [43] with minor modifications. A total of 50 µL of the sample was mixed with 150 µL of CUPRAC reagent, which consisted of equal volumes of 7.5 mM ethanolic 96% neocuproine solution, 10 mM CuCl_2_·H_2_O solution, and ammonium acetate buffer of pH 7.0. The sample absorbance (EC) was read at 450 nm (Multiskan GO, Thermo Fisher Scientific, Waltham, MA, USA) after a 30-min incubation in the dark at room temperature.

#### 2.7.2. Determination of α-Glucosidase Inhibition

The inhibition of α-glucosidase activity was studied on the basis of the method of Sip et al. [44] with minor modifications. A total of 50 μL of 0.1 M phosphate buffer (pH 6.8), 50 μL of the sample, prepared similarly to the solubility test, and 30 μL of α-glucosidase solution (0.5 U·mL^−1^) were added to wells of a 96-well microplate. After 15 min pre-incubation at 37 °C, to start the reaction, 20 μL of 5 mM p-nitrophenyl-α-D-glucopyranoside (pNPG) solution in a 0.1 M phosphate buffer (pH 6.8) as a substrate was added. The plate was then incubated at 37 °C for 20 min, after which, with the addition of 100 μL of sodium carbonate (0.2 M), the reaction was terminated. The absorbance of the product of the reaction (p-nitrophenol) was measured at 405 nm. As a positive control, acarbose, in varying concentrations, was used. The inhibition of enzyme activity was calculated using the equation:

$$\%\;inhibition\;activity=((A_{C}-A_{S})/A_{C})\times 100$$

where *A_C_* refers to the absorbance of the control, which indicates 100% enzyme activity, and *A_S_* represents the absorbance of the tested sample.

### 2.8. Statistical Analysis

The statistical analyses were conducted using the Statistica 13.3 software (StatSoft, Krakow, Poland). The collected data were subjected to a one-way analysis of variance (ANOVA) followed by Duncan’s post hoc test. Statistical significance was determined by a probability level of *p* < 0.05. The outcomes are expressed as mean ± standard deviations.

## 3. Results and Discussion

### 3.1. Molecular Modeling

To determine the optimal amino acids for obtaining co-amorphous systems with GEN, a molecular modeling screening was employed. The results of GEN subjected to docking simulations with the 20 amino acids are presented in Table S1. The resulting systems were evaluated based on their binding energies, and the five most favorable amino acids, namely lysine (LYS), arginine (ARG), tryptophan (TRP), tyrosine (TYR), and glutamine (GLU), were identified. Table 1 presents a summary of the lowest binding energies observed for the systems, as well as the specific interactions between GEN and each amino acid. The interaction between GEN and the five amino acids, which formed the most energetically favorable systems, were visualized and can be seen in Figure S1 of the Supplementary Materials.

Simple molecular models of GEN-amino acid systems confirmed the presence of hydrogen bonds in all cases. Hydrophobic interactions were evident in all complexes except for the GEN–GLU system. Theoretical predictions also suggest the formation of π–π stacking interaction in the GEN–TRP and GEN–TYR systems.

Based on our previous research, it has been established that basic molecular modeling is a useful tool for demonstrating the existence and type of molecular interactions [45,46]. Moreover, it has been demonstrated that the application of molecular modeling techniques facilitates a comprehensive understanding of the interactions transpiring in amorphous systems [47].

### 3.2. Preparation and Identification of Genistein—Amino Acids Systems

#### 3.2.1. X-ray Powder Diffraction

To produce co-amorphous GEN systems with amino acids, we applied the ball milling technique, which results in the breaking of the crystal lattice and conversion of crystalline substance into its amorphous form. Ball milling is an eco-friendly technology frequently used to obtain amorphous systems [48] in an easy, affordable, and repeatable manner.

To characterize the presence of amorphous material, we used XRPD. The diffractogram of pure GEN “as supplied” is shown in Figure S2a. Sharp peaks confirm the crystalline nature of pure GEN. Grinding of GEN without the addition of co-formers failed to produce an amorphous substance under the used process parameters (Figure S2b). Crystal peaks can still be detected; however, the half-width of the peaks became broader, and the peaks have lower intensities than unprocessed GEN, indicating pronounced destruction of the crystal lattice.

**Figure 1:**
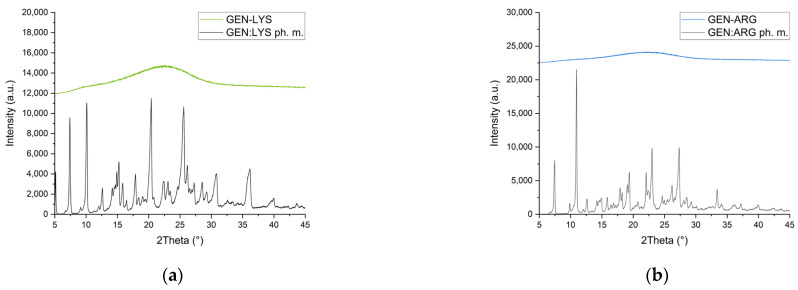
XRPD diffraction patterns of co-amorphous systems of GEN with LYS and the physical mixture (**a**), co-amorphous systems of GEN with ARG, and the physical mixture (**b**).

A good co-former should lead to the amorphization of the active compound, which can be seen as a halo effect on the XRPD diffractogram due to the lack of long-range order. Three of the five amino acids—LYS, ARG (Figure 1a,b, respectively), and TRP selected by the molecular modeling—resulted in the generation of disorder in GEN; however, the reduction in crystallinity was lower for systems with TRP (Figure S3a).

The usefulness of the selected three amino acids as co-formers has been demonstrated in the literature. It was possible to obtain co-amorphous systems by milling LYS with indomethacin [49], mebendazole, simvastatin, furosemide [50], and valsartan [32]. Diffraction peaks in XRPD traces of furosemide, indomethacin [50], naproxen [51], nitrofurantoin, cimetidine, mebendazole [52], and quercetin [53] disappeared after milling with ARG, and TRP has been successfully used to form co-amorphous systems with carvedilol [33], mebendazole, carbamazepine, simvastatin, furosemide and indomethacin [50]. However, in our study, the TRP-containing systems were more crystalline than the LYS and ARG systems with GEN.

Despite the indicated low energies in molecular modeling, TYR and GLU failed to produce co-amorphous systems with GEN (Figure S3b). This is in line with previous studies in which TYR turned out to be a poor co-former for indomethacin and carbamazepine [54]. The results of our experiment support the notion [50] that polar amino acids, such as TYR and GLU, lack effective co-forming properties when used in ball milling. The findings show that, although modeling is helpful for the initial selection of a co-former, it should be supported through empirical observations.

Further characterization and studies were done on the GEN systems with LYS and ARG since XRPD characterized them as amorphous.

#### 3.2.2. Thermogravimetric and Differential Scanning Calorimetry

**Figure 2:**
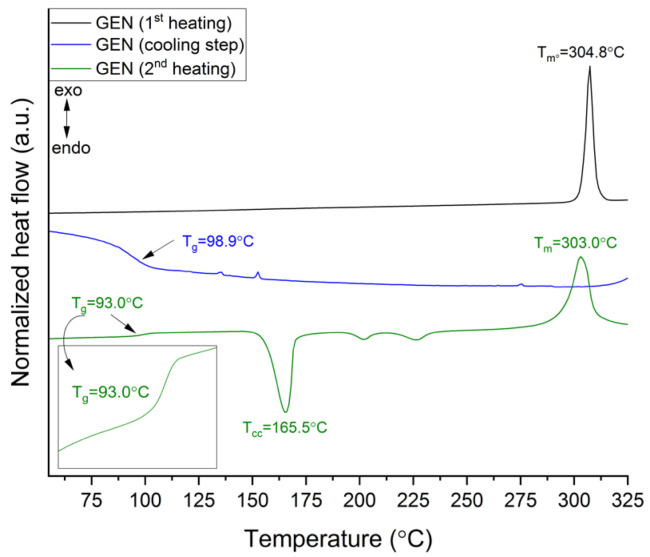
DSC thermograms of GEN.

The thermal stability of GEN and amino acids was determined using the TG method. The results showed that GEN is stable up to a temperature of 310.0 °C, while LYS and ARG are stable up to 215.0 °C and 227.0 °C, respectively (Figure S4). Figure S4 (green line) shows a DSC thermogram of GEN. The melting point of GEN can be seen as a sharp endothermic peak at 304.8 °C. This value is consistent with literature data [22] and indicates the crystalline state of GEN “as supplied”. As GEN is difficult to transform into an amorphous form, no experimental T_g_ of this molecule has been published to date. Based on the DSC thermograms of GEN and poly(D,L-lactic acid) (PDLLA), Buddhiranon et al. [55] hypothesized that a T_g_ of GEN might be lower than the T_g_ of pure PDLLA, which is less than 42 °C. Our analysis enabled us to suggest a T_g_ for GEN. To observe it, heating–cooling modes (see Section 2.4.2) were used. In the cooling step, melted GEN showed a T_g_ at 93.0 °C, whereas in the second heating step, T_g_ was observed at 98.9 °C. Apart from T_g_, cold crystallization (T_cc_ = 165.5 °C), two exothermic effects (T_ee1_ = 201.9 °C and T_ee2_ = 226.4 °C), most likely associated with crystallization, and melting point (T_m_ = 303 °C) were found during the second heating stage (Figure 2).

TG showed ~15% mass loss of GEN at 345 °C (endpoint temperature of melting process in DSC chamber). The percentage of GEN crystallinity after the melting process was determined to be about 60.8%, calculated using the following equation:

$$\%\;\mathrm{Crystallinity}=\frac{\Delta H_{m}-(\Delta H_{cc}+\Delta H_{ee})}{\Delta H_{m{}^{\circ}}}\times 100\%$$

In this equation, the heats of melting (ΔH_m_) and cold crystallization (ΔH_cc_ and ΔH_ee_) are in terms of J·(g °C)^–1^. ΔH_m°_ stands for a reference value and represents the heat of melting in J·(g °C)^–1^ if the GEN was 100% crystalline. Hence, it is suggested that the observed T_g_ is related to the presence of ~40% of the GEN amorphous phase.

The thermal stability of GEN–LYS and GEN–ARG was determined by a TG study and is shown in Figure S5. The first effect seen at approximately 100 °C corresponds to the water loss. Subsequent mass loss is observed above 200 °C, reaching approximately 22–25% loss around 300 °C, a temperature that coincides with the melting point of GEN. For this reason, it is advisable to conduct a DSC analysis up to 200 °C [56].

**Figure 3:**
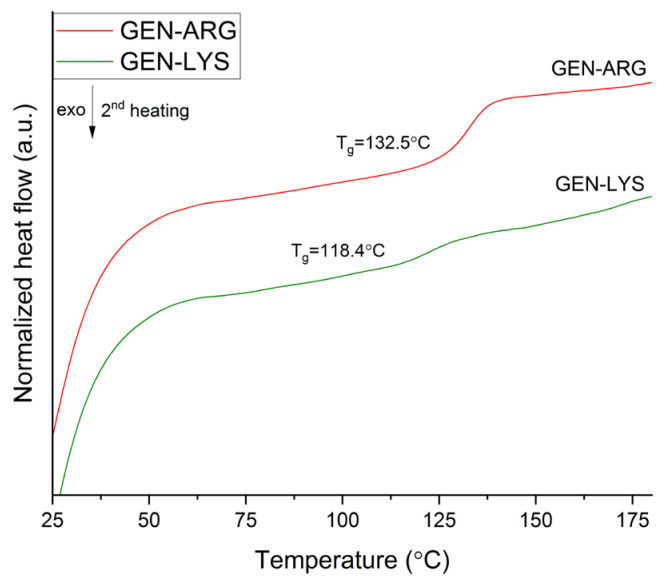
DSC thermograms of GEN–LYS and GEN–ARG co-amorphous systems.

The DSC thermograms of the obtained systems are depicted in Figure 3.

DSC analysis made it possible to observe T_g_ values for both systems. Due to the fact that the T_g_ is a parameter specific to amorphous substances [57,58], it confirmed the findings of an XRPD investigation of GEN in its amorphous state.

The T_g_ is also an important parameter regarding the physical stability of the system. Below the T_g_, molecular mobility is decreased. Hence, the risk of recrystallization is reduced [59]. Since we did not produce pure amorphous amino acids, their T_g_s values were obtained from the literature and are 68 °C and 55 °C for LYS and ARG, respectively [60,61]. The amorphous binary systems had T_g_ values of 118.4 °C for the system with LYS and 132.5 °C for the system with ARG, respectively (Figure 3). Thus, they were higher than the T_g_s of pure GEN and amino acids alone. These results can be expected from the literature findings. Jensen et al. [34] noted that the indomethacin and furosemide systems with TRP had a higher T_g_ than those of the individual components, demonstrating the role of amino acids as antiplasticizers. Huang et al. [32] found similar outcomes when they milled valsartan with ARG, LYS, and histidine. Additionally, these authors state that the rise in the T_g_ of the resulting systems shows the presence of interactions between their constituent parts. Huang et al. [32] also indicate that the systems with the greatest and lowest T_g_ values are arranged in accordance with the presence of variations in the pK_a_ values between the amino acids and the active ingredient. In our study, the same association was found to exist.

Previous studies suggest that the pK_a_ value difference between the co-former and the API, while not the only one, may nonetheless be important for the creation of co-amorphous systems. The likelihood of ionic interactions increases with a pK_a_ value difference >2 or 3 [50]. The protons of the GEN C-7 and C-4’ hydroxyl groups dissociate easily, while the C-5 hydroxyl group’s proton hardly detaches from the group [62]. The value of its first dissociation constant is 7.25 [63]. Thus, for the LYS (pK_a_ of side chain = 10.62 [32]) and ARG (pK_a_ of side chain = 12.48 [32]) systems with GEN, the expected difference in pK_a_ values would suggest ionic interactions. For the TRP (pK_a_ of α-ammonium ion = 9.41 [64]) system with GEN, the difference is lower. These three amino acids appeared to have good co-formability properties to produce co-amorphous systems with GEN. TRP is mentioned as a useful co-former in the Kasten et al. study [50], as it made it possible to create systems with acidic, basic, and neutral drugs. Nevertheless, our research found that TRP was not a suitable co-former for generating a co-amorphous system through milling with GEN.

The experimental T_g_ values obtained for the systems were compared to the theoretical T_g_ values calculated using the Gordon-Taylor (G-T) equation:

$$T_{g,G-T}=\frac{w_{1}T_{g1}+kw_{2}T_{g2}}{w_{1}+kw_{2}}$$

where in the co-amorphous system with LYS, the weight fraction of GEN and LYS components is defined as *w*_1_ = 0.6489 and *w*_2_ = 0.3511, while in system involving ARG, the weight fraction of GEN and ARG components is specified as *w*_1_ = 0.608 and *w*_2_ = 0.392, T_g1_ and T_g2_ is the glass transition temperature of GEN and amino acid, respectively; the constant *k*, which is related to density and expansion coefficient, can be estimated using the Simha-Boyer rule:

$$k=\frac{\rho_{1}\Delta \alpha_{1}}{\rho_{2}\Delta \alpha_{2}}\approx \frac{\rho_{1}T_{g1}}{\rho_{2}T_{g2}}$$

The experimental determination of densities for each component (as described in Section 2.4.2) resulted in the following values: $\rho_{1}$ for GEN is 1.4851 g·cm^−3^ ± 0.0011 g·cm^−3^, and $\rho_{2}$ for amino acids (LYS: 1.1863 g·cm^−3^ ± 0.0008 g·cm^−3^, ARG: 1.3027 g·cm^−3^ ± 0.0027 g·cm^−3^).

According to the G-T equation, the calculated theoretical T_g_ values were 83.6 °C for the LYS binary system and 73.9 °C for the ARG binary mixture. The G-T equation relies on the free volume theory and assumes that there are no specific interactions within the system while also considering the additivity of basic thermo-physical properties. The variations noticed between the experimental and theoretical values can be attributed to the interactions occurring between GEN and amino acids. This is confirmed by the observation of higher experimental T_g_ values compared to T_g_ values predicted by the G-T equation, indicating positive deviations. These deviations may be caused by strong heteronuclear interactions such as hydrogen bonding [65].

#### 3.2.3. Fourier-Transform Infrared Spectroscopy

**Figure 4:**
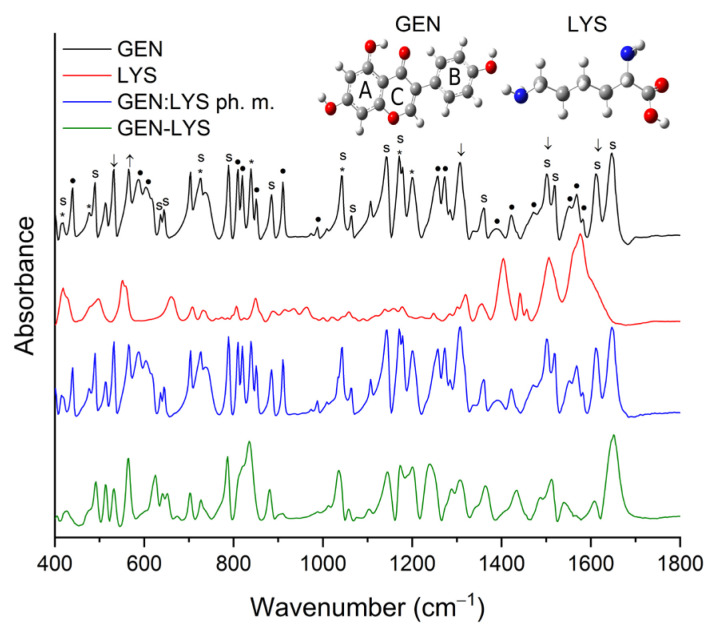
FT-IR spectra in the range of 400–1800 cm^−1^ of GEN (black line), LYS (red line), GEN:LYS physical mixture (blue line), GEN–LYS co-amorphous system (green line).

To provide more information about the potential interactions between GEN and amino acids in the acquired co-amorphous systems, FT-IR analysis was carried out. Weak absorption peaks made it not possible to identify LYS in the physical mixture of GEN:LYS (represented by the blue line in Figure 4 and Figure S6). Crystalline GEN in the physical mixture demonstrated no alterations. As opposed to the crystalline counterpart, amorphous GEN exhibited significant modifications, including the absence of several characteristic peaks within the fingerprint region (650–1800 cm^−1^), as depicted in Figure 4 and Table S2, and changes in the intensity and location of numerous peaks. For example, the peak corresponding to the vibration of the C–C–C group in B-ring, C4′–OH, and C1′–C3 groups at approximately 725 cm^−1^ in the GEN–LYS co-amorphous system underwent alterations in both shape and position and it shifted to 727 cm^−1^. Shifts can also be detected for the peaks at 789/786 cm^−1^ (C–C–C and C4=O), 885/880 cm^−1^ (C–C–C and C–C), 1041/1035 cm^−1^ (C=C in B-ring and O1–C2), 1064/1059 cm^−1^ (O1–C9 and C–C–C), 1141/1144 cm^−1^ (C–H and C7–OH), 1171/1173 cm^−1^ (C–H), 1500/1487 cm^−1^ (C–H, 5–OH and C=C), 1518/1512 cm^−1^ (C=C in B-ring, C–H), 1611/1609 cm^−1^ (C=C in B-ring and C2=C3), 1647/1651 cm^−1^ (C=C and 5-OH) for GEN/GEN–LYS, respectively. In addition, a change in the shape of the bands between 810–850 cm^−1^ and 1075–1330 cm^−1^ is observed. In the first range, crystalline GEN has three well-separated bands with maxima at 810 cm^−1^, 820 cm^−1^ and 839 cm^−1^. In contrast, in the case of GEN–LYS, we observe a single band with an altered shape, which manifests as a single peak with a maximum at 835 cm^−1^ and an edge at about 818 cm^−1^. In the second range, the shape change concerns the bands at 1141 cm^−1^, 1171 cm^−1^, 1200 cm^−1^, 1257 cm^−1^, 1273 cm^−1^. These changes may be the result of a lack of long-lasting structural ordering in the amorphous sample, which leads to wide energy dispersion in the IR spectrum and makes it difficult to separate the individual bands clearly. Additionally, changes in the range of 2700–3500 cm^−1^ observed for the O–H stretching vibration of GEN confirmed the existence of intermolecular hydrogen bonds. Based on the observed changes in the nature of the GEN–LYS spectrum, it is indicated that strong hydrogen bonds are responsible for maintaining the amorphous state of GEN.

**Figure 5:**
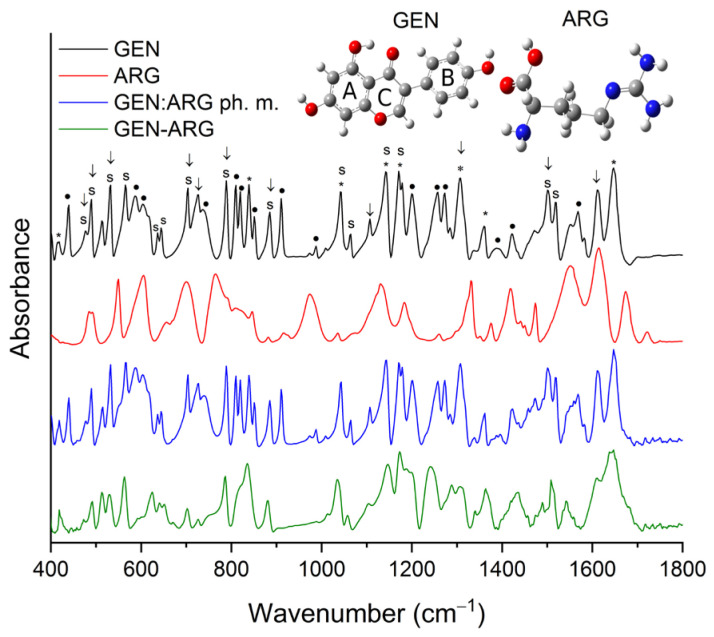
FT-IR spectra in the range of 400–1800 cm^−1^ of GEN (black line), ARG (red line), GEN:ARG physical mixture (blue line), GEN–ARG co-amorphous system (green line).

Crystalline ARG was not detectable in the physical mixture of GEN:ARG (shown by the blue line in Figure 5 and Figure S9) due to its weak absorption peaks. Similar to the physical mixture of GEN–LYS, crystalline GEN in the physical mixture of GEN–ARG exhibited no changes. Conversely, amorphous GEN in the resultant GEN–ARG co-amorphous system displayed considerable changes in comparison to its crystalline form (as depicted by the green line in Figure 5 and outlined in Table S3). For example, changes in location were observed for the peaks at about 490/492 cm^−1^, 532/530 cm^−1^, 565/563 cm^−1^, 603/623 cm^−1^, 644/650 cm^−1^, 704/702 cm^−1^, 789/785 cm^−1^, 885/881 cm^−1^, 1041/1036 cm^−1^, 1064/1057 cm^−1^, 1141/1148 cm^−1^, 1171/1173 cm^−1^, 1500/1488 cm^−1^, 1518/1508 cm^−1^ for GEN/GEN–ARG, respectively. In addition, a change in the shape of the bands between 810–850 cm^−1^ and 1075–1330 cm^−1^ is observed. In the first spectrum, crystalline GEN has three well-separated bands with maxima at 810 cm^−1^, 820 cm^−1^ and 839 cm^−1^. In contrast, in the case of GEN–ARG, we observe a single band with an altered shape, which manifests as a single peak with a maximum at 835 cm^−1^ and an edge at about 818 cm^−1^. In the second range, the shape change concerns the bands at 1141 cm^−1^, 1171 cm^−1^, 1200 cm^−1^, 1257 cm^−1^, 1273 cm^−1^. As with the GEN–LYS system, these changes may be the result of a lack of long-lasting structural ordering in the amorphous sample. Subsequently, alterations in the range of 2700–3500 cm^−1^ observed for the O–H stretching vibration of GEN confirmed the presence of intermolecular hydrogen bonds. The alterations noted in the GEN–ARG spectrum suggest that strong hydrogen bonds play a crucial role in preserving GEN’s amorphous state.

By correlating the FT-IR outcomes with molecular modeling data, it may be inferred that the optimal energetic interactions between GEN and LYS are attributed to the hydrogen bond between the 4’-OH and C4=O groups of GEN and NH_2_ group of LYS, and hydrophobic interaction between the B-ring of GEN and C_β_ atom of LYS. This is consistent with the literature [66], which indicates that the amphiphilic moiety consisting of a phenyl ring with a hydroxyl group exhibits the ability to function as both a hydrogen bond donor and acceptor. Subsequently, it is postulated that the most favorable energetic interactions between GEN and ARG may involve the formation of a hydrogen bond between the 5-OH group of GEN and the NH_2_ group at the C_α_ atom of ARG and hydrophobic interaction between the C3 atom of GEN and C_β_ atom of ARG. Theoretical predictions are consistent with experimental findings, providing evidence for alterations in the GEN spectrum, including shifts and disappearance of several characteristic bands attributed to the 5-OH group.

The results of the FT-IR spectral analysis demonstrate that changes in the overall structure of GEN were induced by the process of amorphization. The stabilization of the amorphous state of the systems was attributed to intermolecular hydrogen bonding and hydrophobic interactions.

#### 3.2.4. Scanning Electron Microscopy

**Figure 6:**
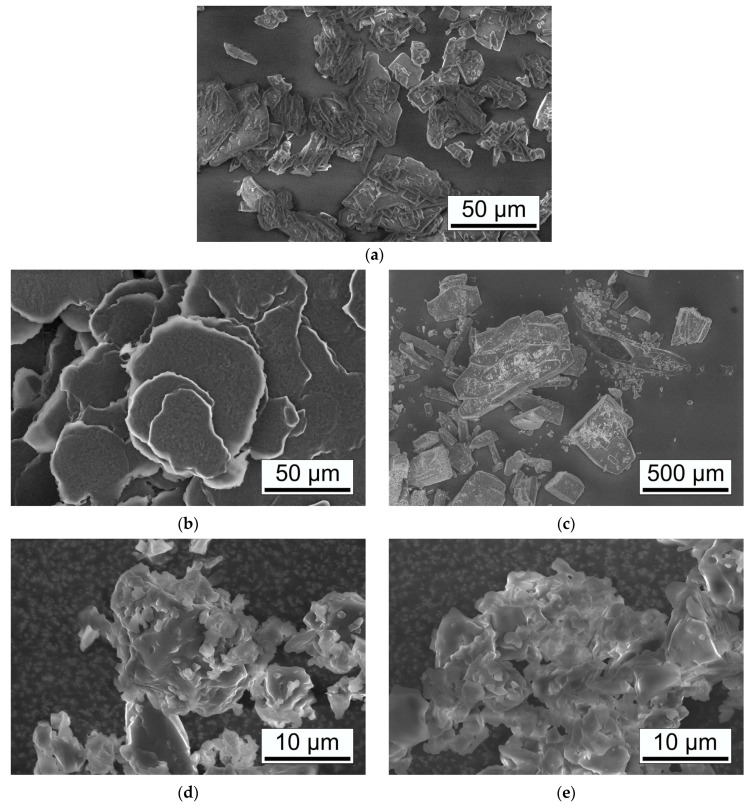
SEM micrographs of GEN (**a**), LYS (**b**), ARG (**c**), GEN–LYS (**d**), and GEN–ARG (**e**) co-amorphous systems. SEM at various magnifications.

Figure 6 shows the morphology of initial and ball-milled powder particles observed by SEM. GEN powder shown in Figure 6a has angular particles with a size between 10 and 50 µm. LYS shown in Figure 6b has flaky in shape particles with sizes ranging between 20 and 50 µm. In contrast to GEN and LYS, ARG powder shown in Figure 6c has the largest angular particles with sizes ranging from 100 to 250 µm, and even particles with sizes of 500–800 µm were observed. The co-amorphous systems GEN–LYS and GEN–ARG, shown in Figure 6d,e, have significantly modified morphology. In both cases, a reduction in the size of particles and the formation of agglomerates with sizes of 5–10 µm were observed.

### 3.3. Physical Stability

Thermodynamic instability of amorphous forms and their propensity to recrystallize present a challenge for their storage and usage. A factor that is particularly favorable to recrystallization is water, as it may decrease the system’s T_g_ and increase molecular mobility. Furthermore, amorphous forms have a strong propensity to absorb water [67]. One of the characteristics of a good co-former is to prevent recrystallization and produce a physically stable system. Stability in co-amorphous systems is primarily brought on by hydrogen bonds and π-π interactions between molecules [68]. Forming molecularly homogenous mixtures in which a compound with a higher T_g_ acts as an anti-plasticizer [69] has an impact on stability as well.

The co-amorphous systems were kept under ambient temperature and humidity. XRPD was used to examine whether the samples still retained their amorphous form, initially fortnightly, then monthly for up to six months. Throughout the storage period, both co-amorphous systems remained physically stable (Figure S11a,b).

The DSC study reveals that both systems exhibit a single T_g_, demonstrating their homogeneity [52], which is considered one of the elements determining the physical stability of amorphous systems. Regarding the impact of T_g_ on the system’s physical stability, it may also be assumed that the more stable would be the GEN–ARG system, which is characterized by a greater T_g_. However, after six months of storage, both systems showed no evidence of recrystallization, as determined by XRPD analysis.

To date, stable systems that use amino acids have been obtained. Combined with indomethacin, ARG provided systems stable for 6 months at 40 °C [54]. When ARG was used as a co-former for (S)-naproxen, a system that remained stable for 10 weeks at 25 °C and 40 °C under dry conditions was obtained [70]. In another study, LYS and ARG provided systems stable in dry conditions at 25 °C for at least 90 weeks with the acidic drugs—furosemide and indomethacin [58]. According to our recent research [45], it was demonstrated that LYS can form stable co-amorphous systems with sinapic acid. Throughout the study, which lasted for six weeks at both 30 °C and 50 °C, there was no evidence of recrystallization.

### 3.4. Physicochemical Properties

#### 3.4.1. Apparent Solubility Studies

The solubility study was carried out in water. The results are presented in Table 2. The apparent solubility of the produced systems was compared to that of crystalline GEN since it was not possible to obtain pure amorphous GEN under the assumed process parameters. GEN solubility was determined to be 0.05 µg·mL^−1^ under the study conditions (see Section 2.6.1). Hence, GEN can be classified as a practically insoluble compound [71], similarly as based on literature reports [19,27]. The improvement in the GEN’s solubility is evident and enables the categorization of GEN as slightly soluble in a system with LYS and very slightly soluble in a system with ARG [71].

Several complementary concepts can be used to explain the observed rise in apparent solubility. As amorphous forms lack long-range ordering and intermolecular interactions are easier to overcome, the improvement in apparent solubility can be attributed to the amorphous form of GEN, which was verified by XRPD and DSC methods in the solid phase. Furthermore, the solubility of GEN is pH-dependent [72]. The pH of the obtained solutions, measured at the end of the study, were 6.48, 9.22, and 9.20 for GEN, GEN–LYS, and GEN–ARG co-amorphous systems, respectively. GEN becomes more soluble as the pH rises. As a result, the alkaline amino acid’s presence can also be connected to improved solubility. In addition, the formation at the molecular level of a homogeneous mixture of GEN with highly soluble compounds such as amino acids may influence solubility. Khanfar et al. [28] noted that when the solubility of the co-former utilized increases, so does the solubility of the co-amorphous system. In our study, a similar association was seen. The improvement in GEN solubility was greater in the co-amorphous system containing LYS, which is more soluble (246.6 mg·mL^−1^) than ARG (195.9 mg·mL^−1^) [32].

Given how much solubility improvement it has provided, the transformation to an amorphous counterpart, using basic amino acids as co-formers, appears to be quite promising in comparison to previous techniques utilized in the literature to increase the solubility of GEN. Chen et al. prepared a complex of κ-carrageenan with GEN. The solubility of amorphous GEN present in the matrix improved 8.9 times at 30 °C, 7 times at 50 °C, and 5 times at 90 °C compared with pure GEN [19]. Sowa et al. reported that cocrystallization of less-soluble GEN with more-soluble caffeine caused an approximately 1.4-times increase in solubility of obtained cocrystal in a 50:50 (*v*/*v*) ethanol–water mixture [18]. Zafar et al. used poloxamer 188 to prepare a ternary inclusion complex with HP-β-CD, reaching a 60.17-fold improvement in aqueous solubility [27].

#### 3.4.2. Powder Dissolution Studies

**Figure 7:**
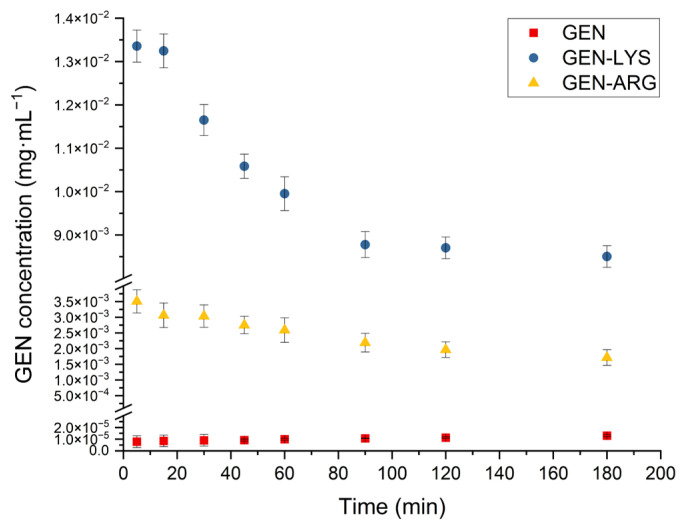
Powder dissolution profiles of crystalline GEN as well as GEN–LYS and GEN–ARG co-amorphous systems.

Powder dissolution profiles of GEN, GEN–LYS, and GEN–ARG co-amorphous systems are depicted in Figure 7.

It can be seen that crystalline GEN has very low solubility. For both co-amorphous systems, an improvement in the apparent solubility of GEN is evident, with a more substantial increase observed in the co-amorphous system containing LYS. However, the maintenance of a high state of supersaturation was unfeasible, resulting in a reduction in the apparent solubility after 30 min. The transformation of the metastable amorphous form into the stable crystalline phase during dissolution is a prominent and well-established complication [73], which can be attributed to the effect of crystallization from the supersaturation state [74] and promoted by the plasticizing effect of water [75]. Nonetheless, taking into account the consensus, the objective is to uphold the utmost achievable supersaturation state [76], the LYS-containing system demonstrated satisfactory results, providing GEN apparent solubility that achieved a plateau at about the 90th min and remained significantly higher than that of crystalline GEN throughout the study.

Apparent solubility achieved for GEN in the system with ARG was lower than in the system with LYS. The observed effect can be attributed to the stronger intermolecular interactions between the GEN and ARG, as deduced from a greater deviation between the experimentally determined T_g_ and the theoretically predicted T_g_ calculated from the G-T equation for the GEN–ARG system. However, solubility higher than the crystalline GEN was maintained throughout the study. A similar effect for a co-amorphous system containing ARG as a co-former was observed by Wu et al. [52]. In their study, furosemide supersaturated concentration decreased slightly after reaching its maximum result, and an extended supersaturation state was then observed. They indicated that this was related to salt formation between ARG and the drug, which translated into an increased chance of inhibiting precipitation.

#### 3.4.3. In Vitro Parallel Artificial Membrane Permeability Assay

Bearing in mind the interplay between apparent solubility and permeability, a PAMPA study for conditions simulating the gastrointestinal tract (GIT) permeability was conducted to evaluate the effect of the obtained systems on the transport of GEN across membranes. It also allows the estimation of passive oral absorption. The study’s findings are shown in Table 3.

The LogP for GEN is equal to 3.04 [77]. It is, therefore, expected that due to its lipophilicity, it will effectively penetrate through membranes. Literature data support this assumption—both the Caco-2 cell model and PAMPA studies have shown its high permeability [78,79,80]. Such a conclusion aligns also with our findings derived from the *P_app_* value in our PAMPA study.

Regarding results obtained for the co-amorphous systems, in the system with ARG, the *P_app_* value was kept above 1 × 10^−6^ cm·s^−1^, indicating that GEN maintained its high permeability in this system. In contrast, the *P_app_* value for GEN in the system with LYS was less than 0.1 × 10^−6^ cm·s^−1^, and should be therefore considered poorly absorbed. The observed decrease in permeability pertains to the system in which greater apparent solubility has been found. The suggestion that increased apparent solubility can result in decreased permeability has been reported in the literature, particularly for techniques that rely on solubilization [81,82,83]. The correlation between solubility and permeability is complex, and high solubility does not always mean high permeability due to various factors such as molecule interactions with cell membranes. The study’s simplified model might also not account for all the factors affecting permeability [84].

In the case of amorphous formulations containing amino acids and ciprofloxacin (CIP), such a relationship was also noted by Mesallati et al. [85]. CIP showed the largest improvement in solubility in amorphous systems with aspartic and glutamic acid, yet it was less permeable compared to pure crystalline CIP. Such a shift could be caused by a rise of ionized CIP in solution. Interestingly, the cysteine and ARG-containing systems did not result in a decrease in CIP permeability. The same observation applies to our co-amorphous GEN–ARG system, in which GEN preserved a permeability classified as high. ARG may also be contributing to this effect via promoting permeation, as suggested by Ruponen et al. [86], who observed enhanced passive membrane permeability for the co-amorphous system of ARG with glibenclamide.

### 3.5. Biological Assays

#### 3.5.1. Antioxidant Activity Determination

**Figure 8:**
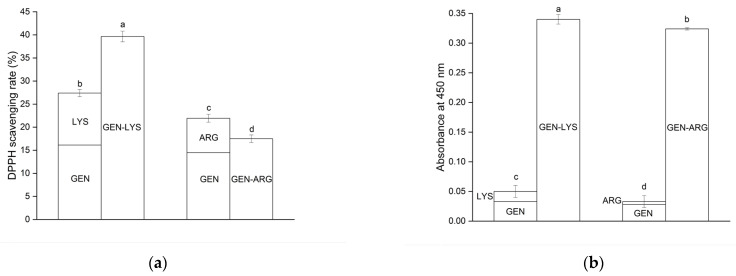
Comparison of antioxidant activity of co-amorphous systems with the sum of antioxidant activities of their constituent components, evaluated using the DPPH (**a**) and CUPRAC (**b**) models. The statistically significant values are denoted by letters from “a” to “d”, where “a” indicates the highest value (*p* < 0.05).

The poor solubility of GEN limits its antioxidant properties [20]. To assess the effect of introducing GEN into the co-amorphous systems on the antioxidant activity, studies using the 2,2-diphenyl-1-picrylhydrazyl (DPPH) radical and CUPric Reducing Antioxidant Capacity (CUPRAC) assay were conducted. The results are presented in Table 4 and Figure 8.

To investigate how improved solubility affects the antioxidant activity of GEN, the concentration of GEN, which was previously determined in a solubility study, was dissolved in dimethyl sulfoxide and subsequently tested for its antioxidant activity.

In the DPPH assay, which is based on the reduction of stable free DPPH radical in the presence of a proton donating antioxidant, the crystalline form of GEN at the concentration achieved during the solubility study displays a scavenging activity of 4.19 ± 0.50% towards the radical. There was an increase in activity observed for the concentrations of GEN in the obtained systems. Compared to crystalline GEN, the activity was 3.8-fold higher for the concentration of GEN present in the LYS system and 3.5-fold higher for the system that included ARG.

The CUPRAC antioxidant capacity assay evaluates an antioxidant’s ability to reduce cupric ions, and the absorbance result increases with greater reduction potential. The antioxidant capacity of GEN in concentrations that were observed in solubility studies surpasses that of pure GEN, as demonstrated by the absorbance values obtained in the CUPRAC assay. At the concentration present in the LYS system, GEN exhibited a 16.5-fold rise in antioxidant capacity, while in the ARG system, it displayed a 14-fold increase compared to its crystalline form (Table 4).

**Table 4:** Antioxidant activity of GEN in crystalline and co-amorphous forms at obtained concentrations.

GEN/GEN in System	Concentration (mg·mL^−1^)	DPPH Assay	CUPRAC Assay
		Radical Inhibition (%)	EC
GEN	5 × 10^−5^ ± 1 × 10^−5^	4.19 ± 0.50 ^c^	0.002 ± 0.003 ^c^
GEN–LYS	1.191 ± 0.161	16.12 ± 0.79 ^a^	0.033 ± 0.002 ^a^
GEN–ARG	0.938 ± 0.014	14.47 ± 0.74 ^b^	0.028 ± 0.001 ^b^

The statistically significant values are denoted by letters from “a” to “c”, where “a” indicates the highest value (*p* < 0.05).

Improved GEN solubility can, therefore, be considered as the reason for the observed enhancement in its antioxidant activity. Such a relationship has already been described in the literature for formulations with other polyphenols [87,88,89,90], as well as for GEN. According to Zafar et al. [27], the enhancement in the solubility of GEN in their ternary inclusion complex was associated with higher radical scavenging activity, and the improvement was concentration-dependent.

However, it should be considered that amino acids may also exhibit antioxidant activity [91,92]. At concentrations equivalent to those present within the systems, LYS and ARG displayed DPPH radical scavenging activity of 11.27 ± 0.12% and 7.46 ± 0.03%, respectively. In the CUPRAC assay, the absorbance values obtained were 0.017 ± 0.001 for LYS and 0.005 ± 0.003 for ARG. Thus, the antioxidant activity of the obtained GEN–LYS and GEN–ARG co-amorphous systems was also investigated. GEN–LYS and GEN–ARG co-amorphous systems inhibited the DPPH radical by 39.64 ± 1.16% and 17.50 ± 0.81%, respectively. This improvement was also notably evident in the CUPRAC method, as the absorbance was 0.340 ± 0.008 and 0.324 ± 0.002 for the GEN–LYS and GEN–ARG co-amorphous system, respectively (Figure 8).

Results showed enhanced antioxidant properties as compared to the activity exhibited by the individual components, except for the GEN–ARG, which did not exhibit superior results in the DPPH test. Based on these findings, it is possible to suggest that GEN and amino acids may engender a synergistic impact regarding their antioxidative potential. Confirmation of the existence of such a relationship can also be found in the literature. Amino acids and phenolic compounds found in Zhenjiang aromatic vinegar can exhibit a synergistic effect by improving each other’s antioxidant properties when they are present together [93].

As such, the observed improvement in antioxidant activity may be attributed to the improved GEN solubility in the obtained co-amorphous systems, in conjunction with the synergistic effect of the amino acids.

#### 3.5.2. Determination of α-Glucosidase Inhibition

GEN has been reported to possess various health-promoting properties. One of its potential pharmacological activities is the inhibition of an enzyme involved in carbohydrate digestion and glucose homeostasis, α-glucosidase [94]. Tadera et al. [95] have indicated that the ability of GEN to inhibit α-glucosidase is affected by certain characteristics of its molecular structure, including the presence of a phenyl group connected to the benzopyran group at position 3 and hydroxylation at position 5. In their study, GEN was observed to exhibit an inhibitory effect on yeast α-glucosidase in a dose-dependent manner, and at a concentration of 200 µM, it provided 93% inhibition of the enzyme. According to Kim et al. [96], GEN exhibited the fifth highest level of inhibitory activity against α-glucosidase out of a total of 21 naturally occurring flavonoids that were examined. In both aforementioned studies, the GEN’s inhibitory activity was assessed after its dissolution in dimethyl sulfoxide as a consequence of its limited solubility in water.

According to our investigation, the presence of GEN in aqueous solutions at feasible concentrations does not display any inhibitory effect on the α-glucosidase, nor do amino acids exhibit any notable activity. However, considerable activity was observed upon the introduction of GEN in co-amorphous systems (Table 5). In the system with LYS, for the achieved GEN water concentration (1.191 mg·mL^−1^), the activity amounted to 99.10 ± 1.24%. Acarbose used as a reference compound at that certain concentration inhibits activity only in 59.63 ± 1.17%. The activity in the system with ARG (in which the concentration of GEN equaled 0.938 mg·mL^−1^) was 94.47 ± 1.59%. At the specified concentration, acarbose exhibits an inhibitory effect solely on 57.14 ± 0.98% of the activity. According to the literature, enhanced solubility was also seen to positively impact the inhibition of α-glucosidase for the poorly soluble polyphenol, hesperidin [97].

The medicinal efficacy of acarbose has been well established. Our experimental findings indicate that the tested systems exhibit greater activity compared to acarbose. Although in vitro α-glucosidase inhibition assay is a valuable tool for initial activity screening, it is essential to recognize its limitations. This assay provides a starting point for development, but further research is crucial to determine the therapeutic potential of the investigated co-amorphous systems.

## 4. Conclusions

The incorporation of amino acids, specifically lysine and arginine, into co-amorphous systems of genistein has been demonstrated to significantly improve the solubility of genistein. The confirmation of the amorphous state of genistein in the lysine and arginine-containing systems was established by X-ray powder diffraction, and their stability was maintained for the entire 6-month storage duration. The biological properties of these systems were further enhanced, with lysine-containing systems displaying the most significant improvement in antioxidant activity. Additionally, these systems demonstrated superior α-glucosidase inhibition relative to acarbose, a standard drug for managing type 2 diabetes. The findings of this study suggest that genistein co-amorphous systems with lysine and arginine have the potential to improve genistein’s pro-health benefits by increasing its solubility and biological activity.

## Figures and Tables

**Table 1:** Binding energies of energy-favorable systems and GEN-amino acid interactions.

		GEN–LYS	GEN–ARG	GEN–TRP	GEN–TYR	GEN–GLU
Binding Energy (kcal·Mol^−1^)		−2.58	−1.96	−1.91	−1.84	−1.83
GEN-amino acid interaction	Hydrogen bonds	O3–H22 O5–H21 H30–O2	O2–H21	H28–O1	H28–O2	O3–H18 H28–O2
	Hydrophobic interaction	✓	✓	✓	✓	none
	π-π stacking (parallel)	none	none	✓	✓	none

“✓” indicates a confirmed interaction and “none” indicates no interaction.

**Table 2:** The results of solubility studies of GEN and obtained systems.

GEN/System	Concentration (mg·mL^−1^)
GEN	5 × 10^−5^ ± 1 × 10^−5 c^
GEN–LYS	1.191 ± 0.161 ^a^
GEN–ARG	0.938 ± 0.014 ^b^

The statistically significant values are denoted by letters from “a” to “c”, where “a” indicates the highest value (*p* < 0.05).

**Table 3:** Apparent permeability coefficient values of GEN in obtained systems.

GEN/System	*P_app_* (cm·s^−1^)
GEN	4.28 × 10^−6^ ± 9.54 × 10^−7 a^
GEN–LYS	8.97 × 10^−7^ ± 1.57 × 10^−8 c^
GEN–ARG	1.13 × 10^−6^ ± 3.13 × 10^−8 b^

The statistically significant values are denoted by letters from “a” to “c”, where “a” indicates the highest value (*p* < 0.05).

**Table 5:** α-glucosidase inhibitory activity of GEN, amino acids, and obtained co-amorphous systems.

GEN/GEN in System	α-Glucosidase Inhibition (%)
GEN	0.00
LYS	0.00
ARG	0.00
GEN–LYS	99.10 ± 1.24 ^a^
GEN–ARG	94.47 ± 1.59 ^b^

The statistically significant values are denoted by letters from “a” to “b”, where “a” indicates the highest value (*p* < 0.05).

## Data Availability

Data are contained within the article.

## Supplementary Materials

The supporting information can be downloaded at https://www.mdpi.com/article/10.3390/pharmaceutics15122653/s1. Table S1. Binding energies of GEN-amino acid systems generated, with the 20 systems ranked in order of increasing energy. The five most energetically favorable systems are highlighted in green. Table S2. Selected characteristic bonds (in cm^–1^) of GEN and GEN-LYS co-amorphous system. Assignments of GEN bands were made based on DFT calculations with application of 6-31G(d,p) basis set and literature [98,99]. Table S3. Selected characteristic bonds (in cm^–1^) of GEN and GEN-ARG co-amorphous system. Assignments of GEN bands were made based on DFT calculations with application of 6-31G(d,p) basis set and literature [98,99]. Table S4. Validation parameters of HPLC-DAD method for concentration determination of GEN. Figure S1. Optimized geometry of GEN, LYS, ARG, TRP, TYR and GLU (images were generated with GaussView) and interactions of GEN with amino acids (images were generated with PyMol). Figure S2. The XRPD diffraction patterns of GEN (a) and ball-milled GEN (b). Figure S3. The XRPD diffraction patterns of ball-milled GEN with TRP (a), with TYR and with GLU (b). Figure S4. TG analysis of GEN, LYS, ARG and DSC thermogram of GEN. Figure S5. TG analysis of GEN-LYS and GEN-ARG co-amorphous systems and DSC thermogram of GEN. Figure S6. FT-IR spectra in the range of 2400-4000 cm^−1^ of GEN (black line), LYS (red line), GEN:LYS physical mixture (blue line), GEN-LYS co-amorphous system (green line). Figure S7. FT-IR experimental results compared with calculations obtained using basis set 6–311G(d,p). Figure S8 Chemical structure with atom numbering of GEN. Figure S9. FT-IR spectra in the range of 2400-4000 cm^−1^ of GEN (black line), ARG (red line), GEN:ARG physical mixture (blue line), GEN-ARG co-amorphous system (green line). Figure S10. Chemical structure with atom numbering of GEN. Figure S11. The XRPD diffraction patterns of stored GEN-LYS (a) and GEN-ARG (b) co-amorphous systems. Figure S12. Chromatograms of GEN for the developed method.
