# Supplementary material for: Genistein Co-Amorphous Systems with Amino Acids: An Investigation into Enhanced Solubility and Biological Activity
Source: Pharmaceutics. 2023 Nov 21;15(12):2653. doi: 10.3390/pharmaceutics15122653 (PMC10747361; doi:10.3390/pharmaceutics15122653)
Supplement: Supplementary file 1 [file pharmaceutics-15-02653-s001.zip › pharmaceutics-2648950-supplementary.pdf]

pharmaceutics

MDPI

Supplementary materials

Genistein Co-Amorphous Systems with Amino Acids: An Investigation into Enhanced Solubility and Biological Activity

Ewa Garbiec [1], Natalia Rosiak [1], Przemysław Zalewski [1], Lidia Tajber [2] and Judyta Cielecka-Piontek [1,*]

[1] Department of Pharmacognosy and Biomaterials, Poznan University of Medical Sciences, 3 Rokietnicka St., 60-806 Poznan, Poland; ewa.garbiec@student.ump.edu.pl (E.G.); nrosiak@ump.edu.pl (N.R.); pzalewski@ump.edu.pl (P.Z.)
[2] School of Pharmacy and Pharmaceutical Sciences, Trinity College Dublin, University of Dublin, Dublin 2, D02 PN40 Ireland; ltajber@tcd.ie
* Correspondence: jpiontek@ump.edu.pl

Table S1. Binding energies of GEN-amino acid systems generated, with the 20 systems ranked in order of increasing energy. The five most energetically favorable systems are highlighted in green.

Amino acid	Type	Binding Energy (kcal·Mol^{-1})
lysine	Basic AA	-2.58
arginine	Basic AA	-1.96
tryptophan	Non-polar AA	-1.91
tyrosine	Polar AA	-1.84
glutamine	Polar AA	-1.83
phenylalanine	Non-polar AA	-1.73
isoleucine	Non-polar AA	-1.69
glutamic acid	Acidic AA	-1.56
leucine	Non-polar AA	-1.55
histidine	Basic AA	-1.5
asparagine	Polar AA	-1.35
methionine	Non-polar AA	-1.33
proline	Non-polar AA	-1.3
alanine	Non-polar AA	-1.12
threonine	Polar AA	-1.12
serine	Polar AA	-1.11
aspartic acid	Acidic AA	-1.07
valine	Non-polar AA	-1.06
cysteine	Polar AA	-0.88
glycine	Non-polar AA	-0.88

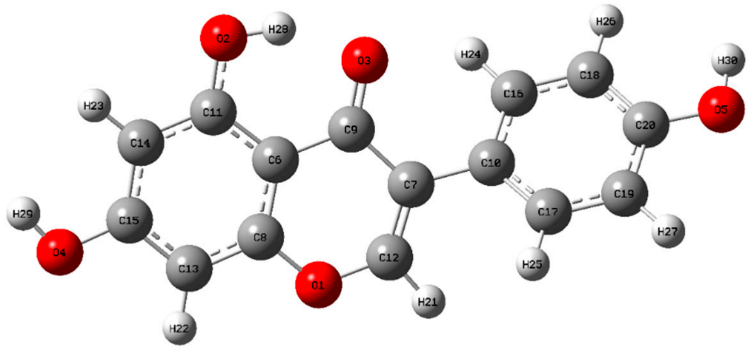

Optimized geometry of the GEN.

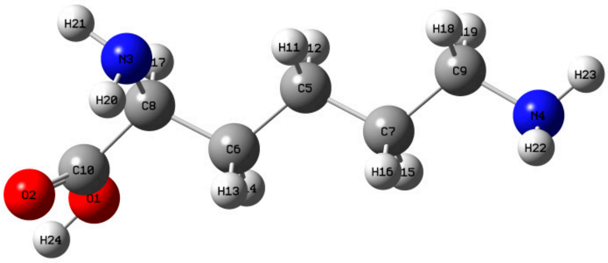

Optimized geometry of the LYS.

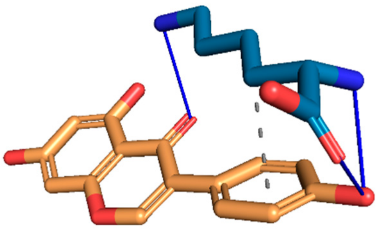

Interaction of GEN with LYS (−2.58 kcal·Mol^{-1}).

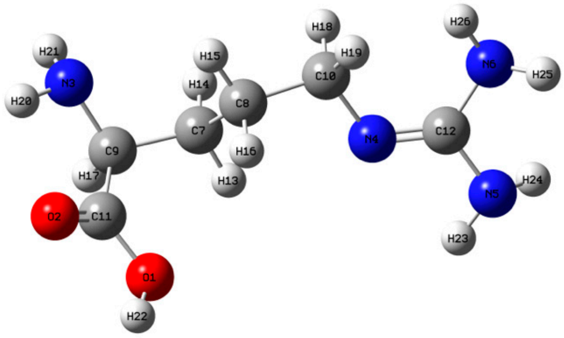

Optimized geometry of the ARG.

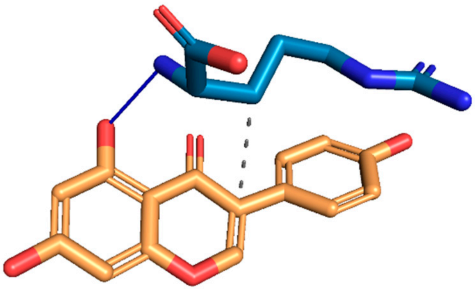

Interaction of GEN with ARG (−1.96 kcal·Mol^{-1}).

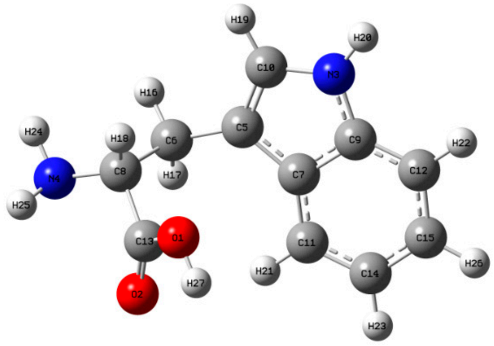

Optimized geometry of the TRP.

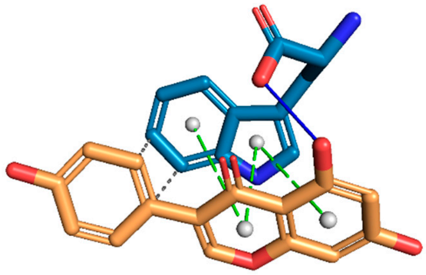

Interaction of GEN with TRP (−1.91 kcal·Mol^{-1}).

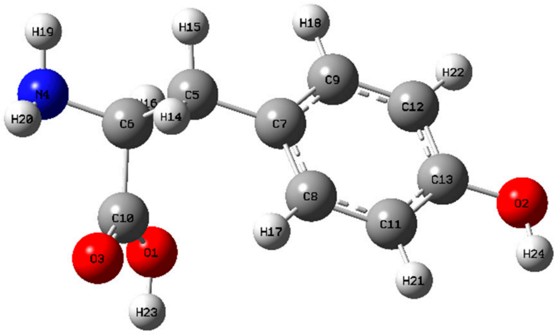

Optimized geometry of the TYR.

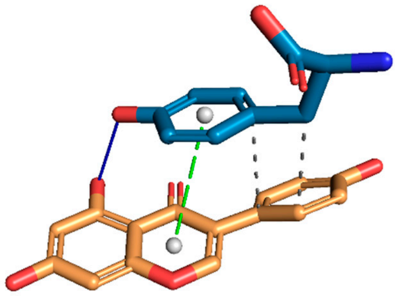

Interaction of GEN with TYR (−1.84 kcal·Mol^{-1}).

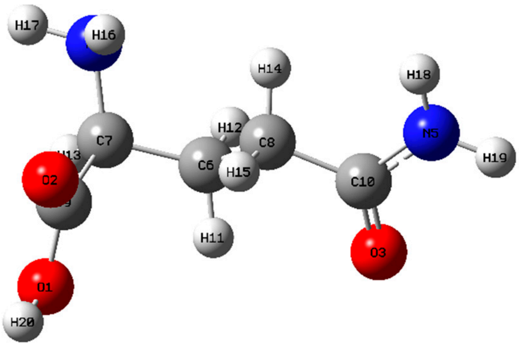

Optimized geometry of the GLU.

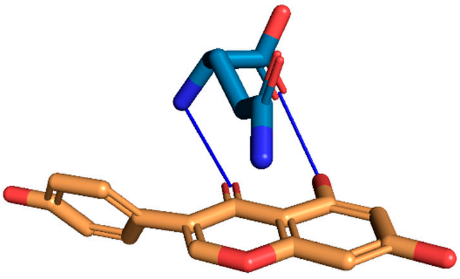

Interaction of GEN with GLU (−1.83 kcal·Mol^{-1}).

Figure S1. Optimized geometry of GEN, LYS, ARG, TRP, TYR and GLU (images were generated with GaussView) and interactions of GEN with amino acids (images were generated with PyMol).

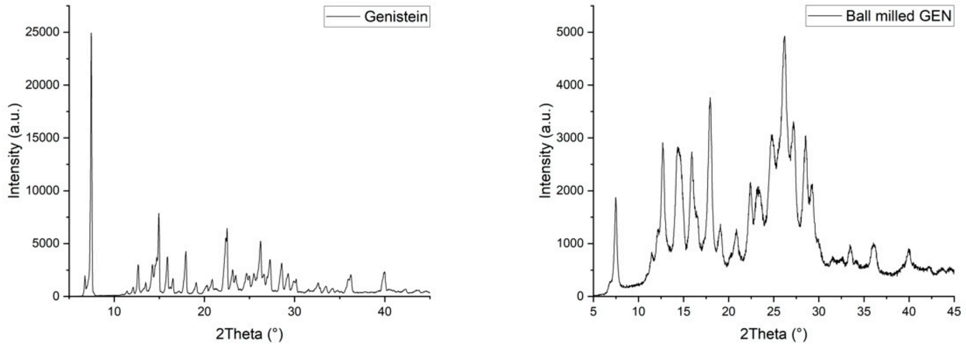

a b

Figure S2. The XRPD diffraction patterns of GEN (a) and ball-milled GEN (b).

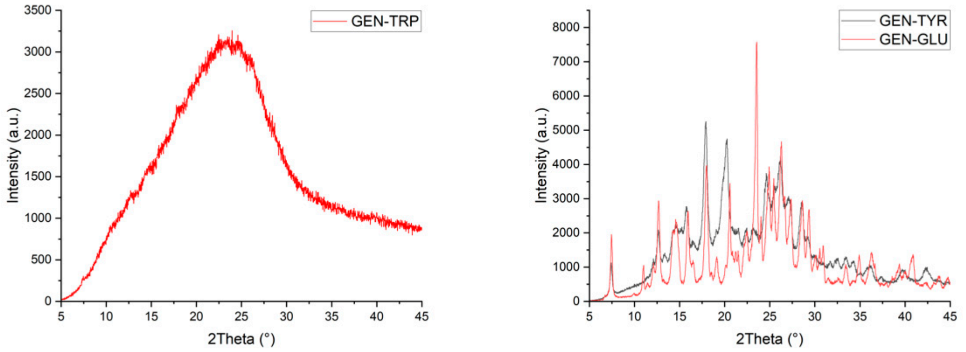

a b

Figure S3. The XRPD diffraction patterns of ball-milled GEN with TRP (a), with TYR and with GLU (b).

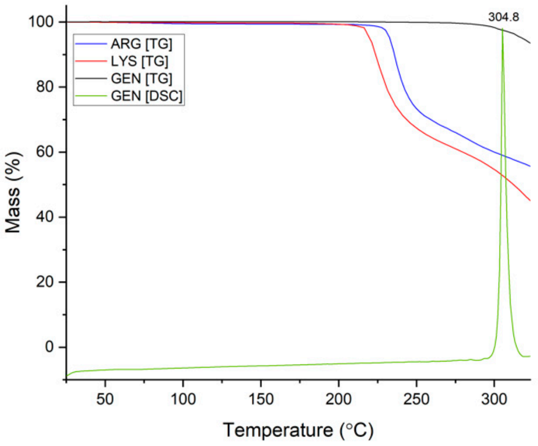

Figure S4. TG analysis of GEN, LYS, ARG and DSC thermogram of GEN.

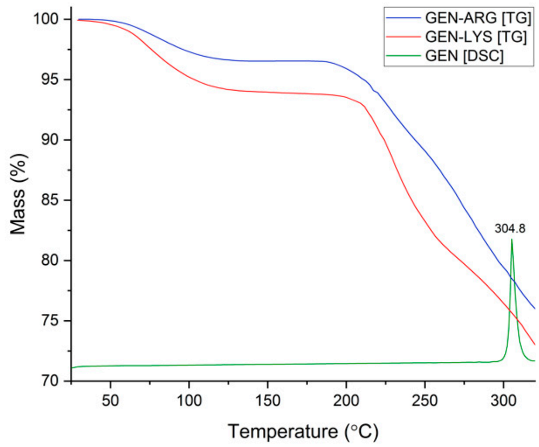

Figure S5. TG analysis of GEN-LYS and GEN-ARG co-amorphous systems and DSC thermogram of GEN.

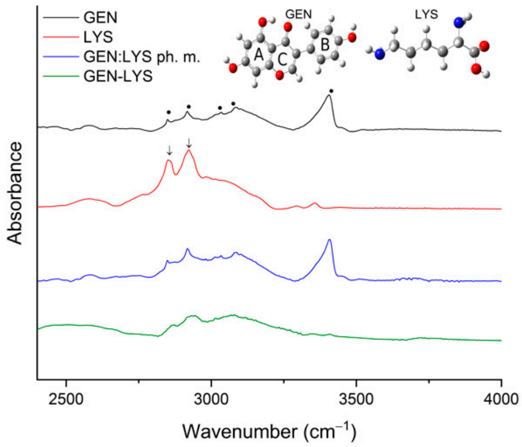

Figure S6. FT-IR spectra in the range of 2400-4000 cm^{-1} of GEN (black line), LYS (red line), GEN:LYS physical mixture (blue line), GEN-LYS co-amorphous system (green line).

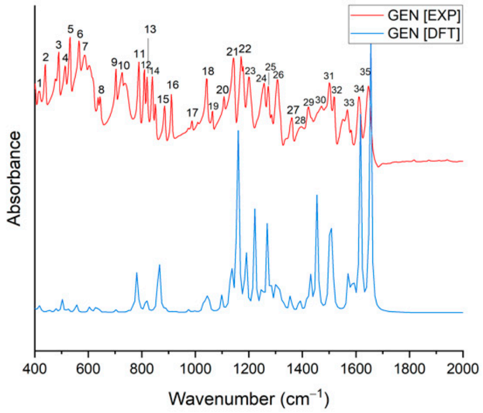

Figure S7. FT-IR experimental results compared with calculations obtained using basis set 6–311G(d,p).

Table S2. Selected characteristic bonds (in cm^{-1}) of GEN and GEN-LYS co-amorphous system. Assignments of GEN bands were made based on DFT calculations with application of 6-31G(d,p) basis set and literature [1, 2].

	GEN	GEN-LYS	
1	416	426*	C–C–C def. in B-ring
2	440	•	C4′–OH in plane b + C–C–C puck in C-ring + C–C–C def. in B- and C-ring
3	490	492	C–C–C def in A-, B- ad C-ring
4	513		C–C–C def. in A- and C-ring
5	532	↓	C4′–OH and C1′–C3 oop b + C–C–C asymmetric torsion def. in B-ring
6	565	↑	C–C–C def in A- and C-ring + C5–OH and C4=O in plane b
7	586	•	C–C–C in plane b and C=C oop b in A-ring + C5–OH oop b
8	644	650	C–C–C def. in B-ring
9	704		C–C–C puck in A-ring + C5–OH oop b
10	725	727*	C–C–C puck in B-ring + C4′–OH and C1′–C3 oop b
11	789	786	C–C–C in plane b in A- and C-ring + C–C in C-ring and C4=O oop b
12	810	•	C–H oop b at A-ring + C–C–C puck in A-ring + C7–OH oop b
13	820	•	C=C in B-ring and C4′–OH oop b + C–C–C def. in B-ring + C–C–C in B- and C-ring in plane b
14	839	*	C–H at B-ring and C4′–OH oop b + asymmetric torsion def. C–C–C in B-ring
15	885	880	C–C–C def. and in plane b in C-ring + C=C oop b in A-ring
16	910	•	C2–H oop b
17	987	•	C=C oop b in A-ring + C–C–C in plane b in A-ring
18	1041	1035*	C=C in B-ring and O1–C2 oop b
19	1064	1059	O1–C9 oop b + C–C–C in plane b in A-ring
20	1107		C–H in plane b in B-ring + C=C oop b in B-ring
21	1141	1144	C–H in plane b at A-ring + C7–OH oop b
22	1171	1173*	C–H in plane b at B-ring
23	1200	*	O1-C2 and C–C in C-ring oop b + C–H in plane b at A-ring
24	1257	•	C3–C1′and O1–C2 oop b
25	1273	•	C4′–OH oop b + C–C–C in plane b in B-ring
26	1305	↓	C=C oop b in A-ring
27	1360	•	C2=C3 oop b + C2–H in plane b
28	1396	•	C=C oop b in A-ring
29	1423	•	C–C oop b in C-ring + 5-OH in plane b
30	1472	•	C=C oop b in A-ring + C4=O oop b
31	1500	1487↓	C–H at A-ring and 5-OH in plane b + C=C oop b in A-ring
32	1518	1512	C=C in B-ring oop b + C–H in plane b at B-ring
33	1568	•	C=C in A-ring and C2=C3 oop b
34	1611	1609↓	C=C in B-ring and C2=C3 oop b
35	1647	1651	C=C s in A-ring + 5-OH def.
36	2849	•	O–H s
37	2918	•	
38	3034	•	
39	3086	•	
40	3406	•	

Legend: * – band shape change, ↓ – band intensity decrease, • – full disappearance of band, b – bending, def. – deformation, oop b – out of plane bending, puck – puckering, s - stretching, A, B, C

– name of GEN's ring. Carbon atom numbers and localization of A, B and C ring in GEN are depicted in Figure S8.

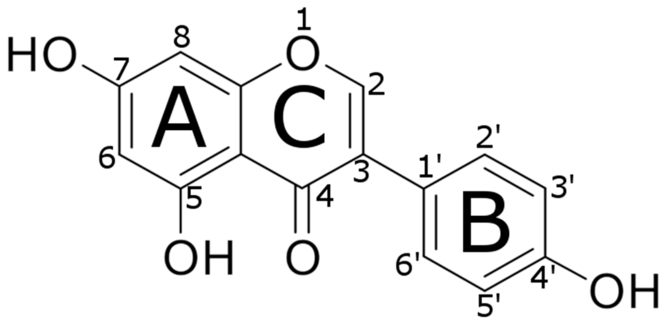

Figure S8 Chemical structure with atom numbering of GEN.

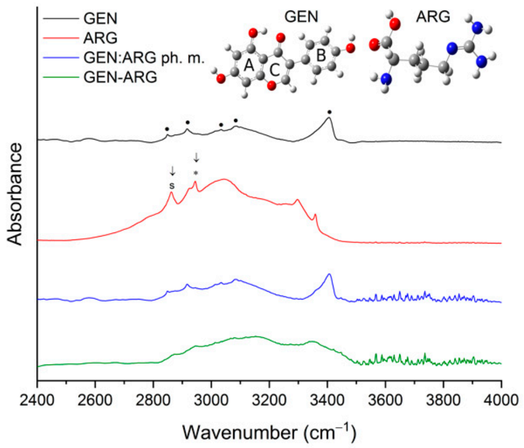

Figure S9. FT-IR spectra in the range of 2400-4000 cm^{-1} of GEN (black line), ARG (red line), GEN:ARG physical mixture (blue line), GEN-ARG co-amorphous system (green line).

Table S3. Selected characteristic bonds (in cm^{-1}) of GEN and GEN-ARG co-amorphous system. Assignments of GEN bands were made based on DFT calculations with application of 6-31G(d,p) basis set and literature [1, 2].

	GEN	GEN-ARG	
1	416	*	C–C–C def. in B-ring
2	440	•	C4′–OH in plane b + C–C–C puck in C-ring + C–C–C def. in B- and C-ring
3	490	492↓	C–C–C def in A-, B- ad C-ring
4	513		C–C–C def. in A- and C-ring
5	532	530↓	C4′–OH and C1′–C3 oop b + C–C–C asymmetric torsion def. in B-ring
6	565	563	C–C–C def in A- and C-ring + C5–OH and C4=O in plane b
7	586	•	C–C–C in plane b and C=C oop b in A-ring + C5–OH oop b
8	644	650	C–C–C def. in B-ring
9	704	702↓	C–C–C puck in A-ring + C5–OH oop b
10	725	↓	C–C–C puck in B-ring + C4′–OH and C1′–C3 oop b
11	789	785↓	C–C–C in plane b in A- and C-ring + C–C in C-ring and C4=O oop b
12	810	•	C–H oop b at A-ring + C–C–C puck in A-ring + C7–OH oop b
13	820	•	C=C in B-ring and C4′–OH oop b + C–C–C def. in B-ring + C–C–C in B- and C-ring in plane b
14	839	*	C–H at B-ring and C4′–OH oop b + asymmetric torsion def. C–C–C in B-ring
15	885	881↓	C–C–C def. and in plane b in C-ring + C=C oop b in A-ring
16	910	•	C2–H oop b
17	987	•	C=C oop b in A-ring + C–C–C in plane b in A-ring
18	1041	1036*	C=C in B-ring and O1–C2 oop b
19	1064	1057	O1–C9 oop b + C–C–C in plane b in A-ring
20	1107	↓	C–H in plane b in B-ring + C=C oop b in B-ring
21	1141	1148*	C–H in plane b at A-ring + C7–OH oop b
22	1171	1173*	C–H in plane b at B-ring
23	1200	•	O1-C2 and C–C in C-ring oop b + C–H in plane b at A-ring
24	1257	•	C3–C1′and O1–C2 oop b
25	1273	•	C4′–OH oop b + C–C–C in plane b in B-ring
26	1305	*↓	C=C oop b in A-ring
27	1360	*	C2=C3 oop b + C2–H in plane b
28	1396	•	C=C oop b in A-ring
29	1423	•	C–C oop b in C-ring + 5-OH in plane b
30	1472		C=C oop b in A-ring + C4=O oop b
31	1500	1489↓	C–H at A-ring and 5-OH in plane b + C=C oop b in A-ring
32	1518	1508	C=C in B-ring oop b + C–H in plane b at B-ring
33	1568	•	C=C in A-ring and C2=C3 oop b
34	1611	↓	C=C in B-ring and C2=C3 oop b
35	1647	*	C=C s in A-ring + 5-OH def.
36	2849	•	
37	2918	•	
38	3034	•	O–H s
39	3086	•	
40	3406	•	

Legend: * – band shape change, ↓ – band intensity decrease, • – full disappearance of band, b – bending, def. – deformation, oop b – out of plane bending, puck – puckering, s - stretching, A, B, C

– name of GEN's ring. Carbon atom numbers and localization of A, B and C ring in GEN are depicted in Figure S10.

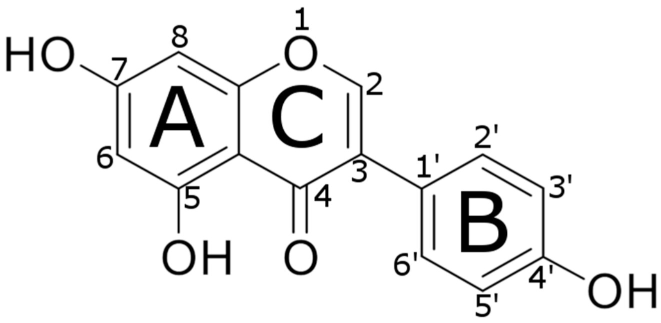

Figure S10. Chemical structure with atom numbering of GEN.

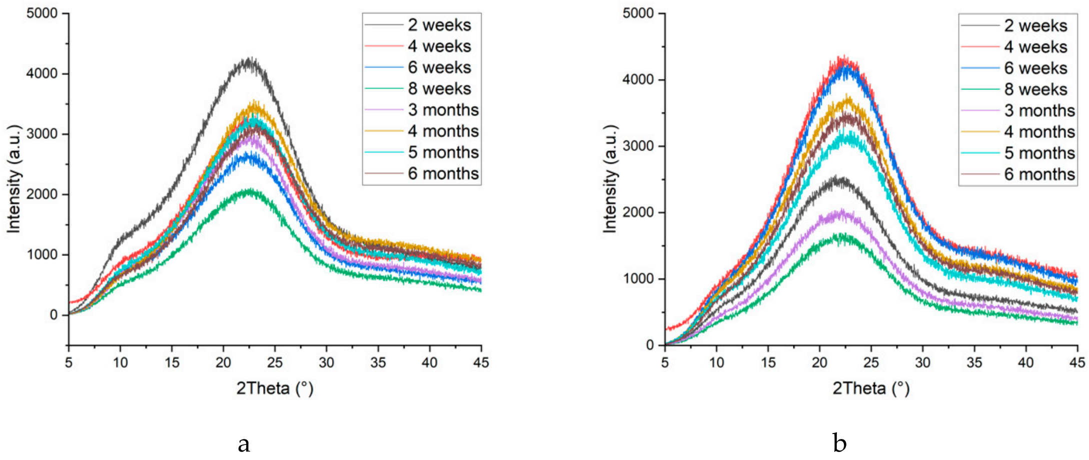

Figure S11. The XRPD diffraction patterns of stored GEN-LYS (a) and GEN-ARG (b) co-amorphous systems.

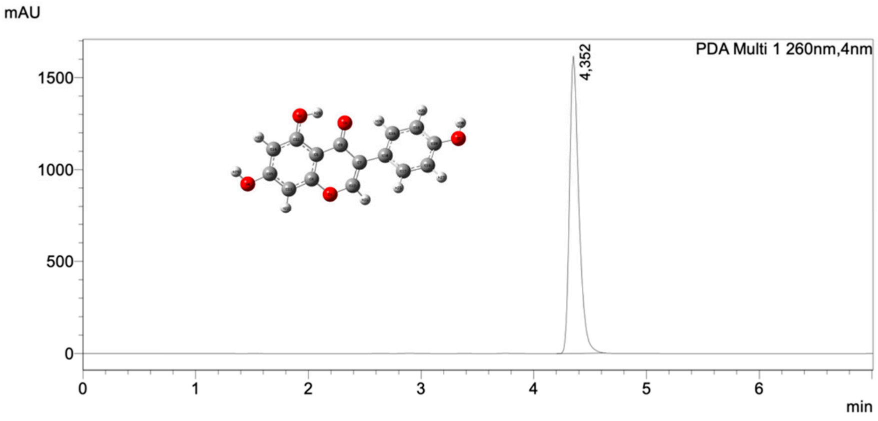

Figure S12. Chromatograms of GEN for the developed method.

Table S4. Validation parameters of HPLC-DAD method for concentration determination of GEN.

Linearity range (mg·mL^{-1})	0.00002-0.3
Correlation coefficient (r)	0.9989
a ± Sa	93757951.63 ± 1126555.206
b ± Sb	insignificant (α=0.05)
LOD (mg·mL^{-1})	0.0132
LOQ (mg·mL^{-1})	0.0401
Retention time (min)	4.352

References:

1. Sekine, R.; Robertson, E. G.; McNaughton, D., Raman, infrared and computational analysis of genistein and its methoxy derivatives. *Vibrational Spectroscopy* **2011,** 57, (2), 306-314.
2. Pawlikowska-Pawlega, B.; Misiak, L. E.; Zarzyka, B.; Paduch, R.; Gawron, A.; Gruszecki, W. I., Localization and interaction of genistein with model membranes formed with dipalmitoylphosphatidylcholine (DPPC). *Biochim Biophys Acta* **2012,** 1818, (7), 1785-93.
